# Supplementary material for: Simulated patient training to improve youth engagement in HIV care in Kenya: A stepped wedge cluster randomized controlled trial
Source: PLOS Glob Public Health. 2023 Apr 19;3(4):e0001765. doi: 10.1371/journal.pgph.0001765 (PMC10115282; doi:10.1371/journal.pgph.0001765)
Supplement: Attachment — Submitted filename: PGPH-D-22-01415 response to reviewers.docx [file pgph.0001765.s004.docx]

**Manuscript ID:** PGPH-D-22-01415

Thank you for the useful review that has aided us in substantially improving this manuscript. Below, we respond to each comment, and we have included a revised clean and tracked changes version of the paper with this submission.

**Reviewer #1**

Thank you for the opportunity to review this manuscript. The authors describe a stepped wedge cluster RCT evaluating the effectiveness of a standardized patient actor training on adolescent engagement in HIV care. The intervention is innovative and underscores important areas for future work.

The study methods are sound and the paper is well-written, but several clarifications could improve its readability. I have suggested revisions and clarifications below, organized by manuscript section.

# Abstract

1. Lines 53-54: could the authors provide specific data/examples, including from Kenya if these data are available? For example, what is the rate of viral suppression among YLHIV in Kenya vs adults, or vs YLHIV in other contexts?

Thank you for this suggestion. We have added a sentence in the introduction with specific estimates for elevated viral load among YLHIV compared to adults in Kenya, “*In Kenya, a 2012-2016 review of national data found elevated viral load among adolescents with HIV 10-19 years was double that of adults (37% vs 13%) (5).”*

2. Line 46: test statistic/p-value for increased satisfaction with care?

We now state, “*Continuous satisfaction with care scores were significantly higher by wave 3 compared to baseline (coefficient=0.38, 95%CI: 0.19-0.58).”*

**Introduction**

3. Lines 75-77: supporting citation?

Apologies for the confusion. That sentence is describing additional details of this study referenced in the previous sentence. We revised the sentence for clarity to state: *They describe feeling conflicted between their own personal feelings, cultural and religious values and beliefs, and their wish to support young people's rights to accessing services*.

**Methods**

4. Line 102/Figure 1: What were the inclusion and exclusion criteria for facilities? I see that 6 facilities were excluded but it is not stated why.

Inclusion criteria are listed under Population and Setting. We reworded this slightly to be more clear about the clinic descriptions being eligibility criteria.

5. Line 131: Which behavioral theories?

Thank you for this question. We have rearranged the conceptual and theoretical descriptions to be more clear. Andersen’s model of healthcare utilization informed our hypothesis that improved provider communication would encourage youth uptake of services (*see lines 92-94 in introduction*). We now specify in the intervention description (*lines 136-138*) that the SP training process is anchored in Kolb’s experiential learning and Bandura’s social learning theories.

6. Line 132: Some more specificity around “concrete experiences, reflection, conventionalization, and active experimentation” would be helpful. What do these terms mean in the intervention context and how were these facilitated?

Apologies for the confusion. We have clarified this sentence (also related to the above comment) to now state, “*SP training is anchored in Kolb’s experiential learning and Bandura’s social learning theories (28, 29); health providers learn through cycles of concrete experiences (mock clinical encounters with SPs), observation, and feedback after the encounters, in a safe and controlled setting.”*

7. Line 135: It’s great that prior work informed the selection of cases. Possible to provide examples of the types of cases that were used, either in text or in a call-out box?

Thank you for this suggestion. We now include a description of cases in the second intervention paragraph.

8. Line 137: How were SP actors selected and trained?

Thank you for this question. We now include this sentence: “*Actors were recruited and auditioned through a casting agency and trained in the SP methodology and the specific cases by an expert trainer and study staff.”*

9. Line 138: Who delivered lectures and facilitated group exercises/debriefs?

We added a sentence in description of the intervention process, “*Lectures and facilitation were delivered by study team clinicians with expertise in adolescent HIV care.”*

**Results**

10. Table 3: Note a question mark in “Patient volume – adolescents”

Thank you for catching this. We have deleted the question mark.

11. Line 225: Did the authors ask about recency of prior AFHS training?

We asked for some information about prior training, however dates and content were entered inconsistently, so we were not able to provide a complete picture of recency.

12. Line 242: Could the authors rephrase the presentation of this aPR (for newly enrolled clients)? It’s not clear what comparison this prevalence ratio represents and since presented immediately following the baseline vs wave 2/3 comparison, unclear if this is comparing change over time or intervention exposure in a subgroup. Similarly for the sensitivity analyses—what were the time periods and comparators for these?

We agree that we should clarify the comparator for each result. Apart for the analysis specifically comparing baseline engagement to wave 2 and 3, the comparison is retention during the unexposed periods (baseline and all waves). In response, we revised the relevant sentences on page 12: *Newly enrolled clients had significantly higher engagement compared to those who had previous gaps in care across all time periods (aPR=1.18, 95%CI=1.05-1.33). Sensitivity analyses restricted to 10-19 year old YLHIV and among all YLHIV, including those continuing in care, were similarly null when comparing exposed and unexposed time periods (aPR=0.91, 95%CI=0.81-1.04 and aPR=0.98, 95%CI=0.90-1.07 respectively).*

**Discussion**

13. Line 282: With the introduction of new MOH/NASCOP guidelines around adolescent friendly services, do the authors feel their intervention effects could have been diluted?

Yes, thank you for this question. There was a large roll out of a national adolescent package of care training in 2015-2016 that may have diluted intervention effects, reflected in overall national improvements in adolescent viral suppression. We now include this in the discussion (lines 297-299).

14. Line 299: I agree with the recommendation to shift training to pre-service settings—is this sort of thing recommended in updated national guidelines?

This is an interesting question. We have not seen pre-service training addressed in national guidelines or training packages, so we did not offer a recommendation related to guidelines.

**Reviewer #2**

The authors present the results of a cluster randomized stepped wedge trial of an intervention to improve heath care worker provision of youth friendly services in Kenya. The manuscript is very well written and clear. A few minor suggestions are provided below.

14. It was a little unclear whether the power was calculated for an increase of 15% from 75% (for an 86% intervention early engagement outcome) or a change from 75% to 90% (a 20% increase). I think it's latter.

The Reviewer is correct that we estimated sample size and power to detect a 15-percentage point difference (75% versus 90%) between control and intervention periods. In response, we revised the sentences in the sample size calculations section on page 8 to read (changes underlined): *The minimum number of records per clinic was estimated for 80% power to detect a 15% difference between control and intervention periods (i.e. 75% versus 90%)…”*

15. It might be helpful to other researchers for the authors to also report the CAC and the IAC used in the power calculation.

Thank you for this suggestion. We used a sample and power calculation spreadsheet for stepped wedge trials developed by Hussey and Hughes (2007). This spreadsheet is available on Professor Hughes’ faculty page: <http://faculty.washington.edu/jphughes/pubs.html>

The spreadsheet does power calculations for a model with a random intercept only, which allows for an estimation of the intra-cluster correlation coefficient (ICC). The model assumed an ICC of 0.25. Therefore, we did not estimate a cluster autocorrelation (CAC) or and the individual autocorrelation (IAC), which are more relevant for cohort studies. In response, we revised the sentence on page 8 to clarify we used a random intercept model: “*The methods described by Hussey and Hughes (33) based on a model with a random intercept were used to determine power assuming an evaluation with 24 clusters and 5 time points (including baseline)…”*

16. The authors did a nice job of justifying stepped wedge over regular cluster randomized trial. Might be good for readers if you also list other advantages to the the stepped wedge other than lack of feasibility of initiating the intervention at a large number of clinics at the same time like minimally detectable difference, number of clusters needed, or the size of the clusters needed?

We agree with this suggestion, and, in response, we have added a sentence about the potential gains in statistical power using a stepped wedge design compared to a parallel design on page 5.

17. It's unfortunate that covid impacted ascertainment of outcomes in the final wave. The authors should be commended for reporting null findings so transparently.

Thank you for this comment.
